# Increase in leaf organic acids to enhance adaptability of dominant plant species in karst habitats

**DOI:** 10.1002/ece3.7832

**Published:** 2021-06-29

**Authors:** Songbo Tang, Jianfeng Liu, Hans Lambers, Lingling Zhang, Zhanfeng Liu, Yutong Lin, Yuanwen Kuang

**Affiliations:** ^1^ Key Laboratory of Vegetation Restoration and Management of Degraded Ecosystems South China Botanical Garden Chinese Academy of Sciences Guangzhou China; ^2^ Heshan National Field Research Station of Forest Ecosystem South China Botanical Garden, Chinese Academy of Sciences Guangzhou China; ^3^ College of Resources and Environment University of Chinese Academy of Sciences Beijing China; ^4^ Key Laboratory of Tree Breeding and Cultivation of State Forestry Administration Research Institute of Forestry Chinese Academy of Forestry Beijing China; ^5^ School of Biological Sciences University of Western Australia Perth WA Australia; ^6^ Department of Plant Nutrition College of Resources and Environmental Sciences National Academy of Agriculture Green Development Key Laboratory of Plant–Soil Interactions Ministry of Education China Agricultural University Beijing China

**Keywords:** adaptation, calcium, Guizhou, mineral, nonkarst, nutrients

## Abstract

Estimation of leaf nutrient composition of dominant plant species from contrasting habitats (i.e., karst and nonkarst forests) provides an opportunity to understand how plants are adapted to karst habitats from the perspective of leaf traits. Here, we measured leaf traits—specific leaf area (SLA), concentrations of total carbon ([TC]), nitrogen ([TN]), phosphorus ([TP]), calcium ([Ca]), magnesium ([Mg]), manganese ([Mn]), minerals ([Min]), soluble sugars, soluble phenolics, lipids, and organic acids ([OA])—and calculated water‐use efficiency (WUE), construction costs (CC), and N/P ratios, and searched for correlations between these traits of 18 abundant plant species in karst and nonkarst forests in southwestern China. Variation in leaf traits within and across the abundant species was both divergent and convergent. Leaf [TC], [Ca], [Min], [OA], and CC were habitat‐dependent, while the others were not habitat‐ but species‐specific. The correlations among [TN], [TP], SLA, [TC], CC, [Min], WUE, [OA], and CC were habitat‐independent, and inherently associated with plant growth and carbon allocation; those between [CC] and [Lip], between [Ca] and [Mg], and between [Mg] and [WUE] were habitat‐dependent. Habitat significantly affected leaf [Ca] and thus indirectly affected leaf [OA], [Min], and CC. Our results indicate that plants may regulate leaf [Ca] to moderate levels via adjusting leaf [OA] under both high and low soil Ca availability, and offer new insights into the abundance of common plant species in contrasting habitats.

## Graphical abstract

Variation in leaf traits within and across the common tree species was both divergent and convergent, and the causal effects of leaf traits were habitat‐dependent or habitat‐independent. Habitat had significant effects on leaf [Ca] and thus indirectly affected leaf [OA], [Min], and CC. Our study revealed leaf [OA] plays an important role in adjusting leaf concentrations and enhances adaptability of dominant plant species in karst habitats, offering new insights into the abundance of common tree species in contrasting habitats, which would be useful in karst forest restoration.

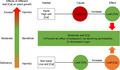

## 1 INTRODUCTION

Karst is a unique ecological system, which is defined as a landscape formed by dissolution of soluble rocks with rocky soils, caves, sinkholes, and lacking surface stream (Geekiyanage et al., 2019; Williams, 2008). Karst habitats are fragile and vulnerable, with high concentrations of calcium ([Ca]) and high pH in their shallow soils (Wei et al., 2018). One of the largest karst ecosystems is located in subtropical mountainous regions of southern and southwestern China (Yuan, 1991), exhibiting remarkably high species richness and endemism, contributing significantly to the floristic diversity of China (Wei et al., 2018), due to the fine‐scale heterogeneity of hydrogeology, topography, and associated water availability influenced by a monsoon climate (Guo et al., 2017). Being important hot spots of biodiversity and endemism (Clements et al., 2006), the karst ecosystems in China are under threats from human disturbance and global change which weaken their stability and accelerate rocky desertification (Lian et al., 2015; Tian et al., 2017).

A variety of plant functional traits have been considered to be related to dynamics of plant communities and functions of forest ecosystems (Boukili & Chazdon, 2017; He et al., 2019; Kunstler et al., 2016). Some plant traits are associated with factors that drive plant diversity and community assembly (Adler et al., 2013; Kunstler et al., 2016); similarity of leaf traits may increase competition among coexisting dominant tree species (Kraft et al., 2015). Many important leaf traits are used to assess plant adaptability and growth in different environments. For example, a high specific leaf area (SLA, Balachowski & Volaire, 2018; Hamann et al., 2018; Lambers & Poorter, 1992) and high leaf nutrient concentrations (Lambers & Poorter, 1992; Zhang et al., 2018) reflect a high capacity for plant growth. Some plant chemicals (secondary metabolites) are helpful to enhance adaptability to stressful environments. Soluble phenolics (SP, Karabourniotis et al., 2014) are related to plant defense under biotic stresses; soluble sugars (SS) serve as osmotic solutes to acclimate to water deficits (Galiano et al., 2017; Kuang et al., 2017); organic acids (OA) are important to sustain cellular functions under drought (Farooq et al., 2009).

The coordination among different leaf traits, which are shaped by evolution (Firn et al., 2019), allows plant adaptation to diverse habitats (Ahrens et al., 2020; Firn et al., 2019; Gratani & Bombelli, 2000; Moreira & Pearse, 2017; Wright et al., 2004, 2005). Correlations among leaf traits are used to interpret the biodiversity–ecosystem functions (Cronin & Schoolmaster, 2018; Schoolmaster et al., 2020) and can be used to track how plants respond to environmental change (Cronin & Schoolmaster, 2018). For example, plants can decrease SLA to increase water‐use efficiency (WUE) in habitats with low water availability (Maxwell et al., 2018) and synthesize OA to abate the adverse effects of excess leaf [Ca] (Kinzel, 1983).

Comparison of the differences in leaf traits and their effects on dominant plant species from contrasting habitats gives an opportunity to understand how dominant plants adapt to different habitats (Geekiyanage et al., 2018). Soil properties, such as soil pH values, water availability, and Ca concentrations, significantly differ between karst and nonkarst habitats (Hao et al., 2015), and these can substantially affect plant growth (Burstrom, 1968; Kinzel, 1983). However, there are a few plant species abundant in both karst and nonkarst forests in southwestern China (Zhu et al., 1998), despite species composition being notably different between the habitats (Fu et al., 2015). In this study, we measured or calculated 15 leaf traits including SLA, concentrations of leaf total carbon ([TC]), nitrogen ([TN]), phosphorus ([TP]), magnesium ([Mg]), manganese ([Mn]), minerals ([Min]), lipids ([Lip]), soluble sugars ([SS]), [SP], [Ca], [OA], leaf construction costs (CC), water‐use efficiency (WUE), and N/P ratios of 18 abundant plant species common to both karst and nonkarst forests in southwestern China. We aimed to test whether (a) leaf traits and their correlations may differ between the two habitats owing to the differences in soil characteristics (Donoghue, 2008; Mori et al., 2019); and (b) leaf traits acclimate to the unique soil parameters, for example, high soil [Ca] and pH values (Hao et al., 2015).

## 2 MATERIALS AND METHODS

### 2.1 Study site, species, and sampling

This study was conducted in Guizhou Province of southwestern China (103°36′–109°35′E, 24°37′–29°13′N), which has a typical karst distribution accounting for approximately 74% of its total area (Zhu, 1993). Characterized by a plateau monsoon humid climate, Guizhou has a mean annual temperature and a mean annual precipitation of 15.5°C and 1,400 mm, respectively, and has typical subtropical karst forests (Tian et al., 2017). Soils in karst forests in this province are generally developed from dolomite and/or limestone, with pH values varying from 6.3 to 7.8 (Wang et al., 2004), and soil [Ca] of 10.6 ± 6.3 mg/g (Zhang et al., 2014). In this study, the karst and nonkarst forests were uniformly selected, with soils developed from limestone and from granite, respectively.

Leaves of 18 common plant species abundant in both karst and nonkarst forests throughout this province (Huang et al., 1988) (Table 1) were sampled for analysis. Each species was sampled from six forests (three karst and three nonkarst). A minimum of three mature trees per species were sampled per forest. At least 10 mature, fully expanded, and healthy leaves were collected per tree. To minimize the influence of tree age, the individual trees of the species were of similar accounts of growth rings, which was determined by tree core extracted using an increment borer (Ф 5.15 mm, Haglöf, Sweden), in both karst and nonkarst habitats. After sampling, the leaves were stored in ice bags and transported back to the laboratory.

**TABLE 1:** The 18 dominant plant species abundant in both karst and nonkarst habitats throughout Guizhou Province. Species names follow Flora of China, available online at www. efloras.org

Species	Family	Leaf type	Life form
*Broussonetia papyrifera* (Linnaeus) L'Heritier ex Ventenat	Moraceae	Deciduous	Tree
*Celtis sinensis* Pers.	Ulmaceae	Deciduous	Tree
*Debregeasia orientalis* C. J. Chen	Urticaceae	Deciduous	Shrub
*Hovenia acerba* Lindl.	Rhamnaceae	Deciduous	Tree
*Camptotheca acuminata* Decne.	Nyssaceae	Deciduous	Tree
*Clerodendrum mandarinorum* Diels	Verbenaceae	Deciduous	Shrub
*Liquidambar formosana* Hance	Hamamelidaceae	Deciduous	Tree
*Ligustrum lucidum* Ait.	Oleaceae	Evergreen	Tree
*Platycarya strobilacea* Sieb. et Zucc.	Juglandaceae	Deciduous	Tree
*Populus adenopoda* Maxim.	Salicaceae	Deciduous	Tree
*Robinia pseudoacacia* L.	Fabaceae	Deciduous	Tree
*Betula luminifera* H. Winkl.	Betulaceae	Deciduous	Tree
*Litsea cubeba* (Lour.) Pers.	Lauraceae	Deciduous	Shrub
*Lindera glauca* (Sieb. et Zucc.) Bl.	Lauraceae	Deciduous	Shrub
*Litsea mollis* Hemsl.	Lauraceae	Deciduous	Shrub
*Quercus fabri* Hance	Fagaceae	Deciduous	Tree
*Triadica sebifera* (Linnaeus) Small	Euphorbiaceae	Deciduous	Tree
*Toxicodendron vernicifluum* (Stokes) F. A. Barkl.	Anacardiaceae	Deciduous	Tree

### 2.2 Leaf trait measurements

In the laboratory, the leaves were cleaned and divided into two parts. One part was used to measure leaf area (Li–COR LI‐3000C, Inc., Lincoln, Nebraska, USA) and then dried to constant weight (65℃ for 72 hr) for SLA calculation. The other part was freeze‐dried, ground, and then used for chemical analyses. Leaf [TC] and [TN] were measured with an elemental analyzer (Isoprime 100, Elementar Isoprime, South Manchester, UK). Leaf [TP] was determined via molybdenum–antimony colorimetry after digestion by sulfuric acid (Murphy & Riley, 1962).

The concentrations of chemical compounds, including leaf [OA], [Min], [TSC], [Lip], [SS], and [SP], were measured according to Poorter et al. (1997) and Blainski et al. (2013). Briefly, a part of leaf powder, about 1.0 g, was extracted with a mixture of chloroform:methanol:water (2:2:1; v:v:v). The chloroform phase was used to determine leaf [Lip] from the residue weighed after evaporation (Poorter et al., 1997). The water/methanol phase was used to determine [SS] and [SP] using the anthrone and Folin–Ciocalteu method, respectively (Poorter et al., 1997). Leaf [lignin] was determined after chloroform:methanol:water extraction and 3% HCl extraction (Poorter et al., 1997).

Concentrations of leaf ${\text{NO}}_{3}^{‐}$ ([${\text{NO}}_{3}^{‐}$]) were determined according to Cataldo et al. (1975). Another part of leaf powder, about 0.10 g, was combusted in a muffle furnace at 550°C for 6 hr. The ashes after combusted consist of minerals (all mineral nutrients in leaves), oxides (derived from OA), and nitrate (Poorter et al., 1997). Leaf ash concentrations ([Ash]) and ash alkalinity were determined acidimetrically. Then, we calculated leaf [OA] and [Min] based on[${\text{NO}}_{3}^{‐}$], [Ash], and ash alkalinity via the following equations:

$$\left[\text{OA}\right]=(\text{Ashalkalinity}‐\left[{\text{NO}}_{3}^{‐}\right])\times 62.1$$

$$\left[\text{Min}\right]=\left[\text{Ash}\right]+\left[{\text{NO}}_{3}^{‐}\right]‐\text{Ashalkalinity}\times 30.$$

Leaf CC was calculated according to Poorter et al. (1997):

$$\text{CC}=\left(‐1.041+5.077\times \left[\text{TC}\right]\right)\times \left(1‐\left[\text{Min}\right]\right)+\left(5.325\times \left[\text{TN}\right]\right).$$

Water‐use efficiency of the plant species was calculated based on leaf δ^13^C values (Ehleringer & Cerling, 1995; Farquhar et al., 1982), which were determined using a mass spectrometer (Thermo Finnigan, North Pod Waltham, Massachusetts, USA). Leaf [Ca], [Mg], and [Mn] were determined by atomic absorption spectroscopy (ContrAA700, Analytik Jena AG, Jena, Germany) after digestion using a Microwave Reaction System (Multiwave 3000, Anton Paar, Graz, Austria).

### 2.3 Statistical analyses

All statistical analyses were conducted using R software (version 4.0.2). The units and key statistic summary of each leaf trait are provided in Table 2. Prior to multivariate analysis, the traits were checked for approximate normality (Shapiro–Wilk test). Those that did not follow normality were log_10_‐ ([TP], SLA, [Ca], [Mg], [Min], [SP]), square‐ ([SS], [OA]), or box‐cox– (CC, [TC]) transformed, and then, all leaf traits standardized to a mean of 0 and *SD* of 1. Differences in leaf traits between the habitats were tested using general linear mixed effect models (GLMEMs), with species and individuals as the random effects (Crawley, 2007), and determined using Tukey's HSD post hoc tests and conducted by the lsmeans function in lsmeans package after processing GLMEMs (Lenth, 2016). The correlations between leaf traits across the habitats were estimated by the pc function and idaFast function in R package pcalg (Kalisch et al., 2012). Briefly, we used the pc function to estimate the equivalence class of a directed acyclic graph (DAG) based on the PC algorithm; then, we used the idaFast function to calculate the coefficient of each pathway in DAG. Pearson's coefficients (*r*) were calculated to test the correlations of the traits between the habitats. We tested the paths of effects of habitat on those traits that differed between habitats via structural equation model (*SEM*) by the sem function in lavaan package. Briefly, we built an a priori model using these leaf traits affected by habitat. After running the a priori model, all nonsignificant paths were removed (*p* > .05) and we ran this new model again. The ratio of chi‐square to degrees of freedom (chi‐square/DF, ≤ 2, *p* > .05), comparative fit index (CFI, ≥0.95), and root mean squared error of approximation (RMSEA, 0 ≤ RMSEA ≤ 0.05) were used to assess the goodness of the final model when chi‐square/DF ≤2 (*p* > .05) (Schermelleh‐Engel et al., 2003). The significance was set at *p* < .05.

**TABLE 2:** Abbreviations, units, means, standard deviation (*SD*), minimum, and maximum values of each leaf trait in this study

Leaf trait	Abbreviation	Unit	Statistical summary [mean ± *SD* (min–max)]	
			Karst	Nonkarst
Leaf carbon concentration	[TC]	mg/g	454 ± 28 (372–499)	459 ± 37 (288–504)
Leaf nitrogen concentration	[TN]	mg/g	25.5 ± 6.19 (8.40–37.7)	25.5 ± 6.43 (15.2–46.8)
Leaf phosphorus concentration	[TP]	mg/g	1.40 ± 0.42 (0.64–2.23)	1.50 ± 0.49 (0.70–2.61)
Leaf *N* to P ratio	*N*/P	unitless	19.0 ± 4.73 (7.10–29.6)	18.1 ± 4.77 (9.31–31.2)
Leaf calcium concentration	[Ca]	mg/g	5.13 ± 3.63 (0.71–13.5)	3.68 ± 2.56 (0.59–11.7)
Leaf manganese concentration	[Mg]	mg/g	0.57 ± 0.38 (0.12–1.74)	0.56 ± 0.32 (0.11–1.69)
Leaf magnesium concentration	[Mn]	mg/g	0.20 ± 0.42 (0.01–2.55)	0.29 ± 0.47 (0.00–2.34)
Leaf mineral concentration	[Min]	mg/g	37.2 ± 24.0 (6.24–104)	32.1 ± 24.9 (3.48–117)
Leaf lipid concentration	[Lip]	mg/g	79.2 ± 21.5 (25.2–145)	78.2 ± 23.5 (2.90–139)
Leaf soluble phenolic concentration	[SP]	mg/g	42.6 ± 33.3 (4.80–134)	40.1 ± 34.6 (7.29–148)
Leaf soluble sugar concentration	[SS]	mg/g	114 ± 65.0 (0.86–345)	102 ± 64.1 (8.03–283)
Leaf organic acid concentration	[OA]	mg/g	105 ± 43.1 (8.76–192)	88.8 ± 44.2 (23.8–209)
Specific leaf area	SLA	m^2^/kg	14.9 ± 4.28 (6.26–23.8)	15.2 ± 4.05 (8.27–25.2)
Water‐use efficiency	WUE	μmol/mol	56.9 ± 16.4 (23.9–90.6)	52.9 ± 15.3 (16–83.2)
Leaf construction cost	CC	g glucose/g	1.34 ± 0.15 (0.90–1.56)	1.38 ± 0.20 (0.49–1.65)

## 3 RESULTS

### 3.1 Variations of leaf traits

**FIGURE 1:**
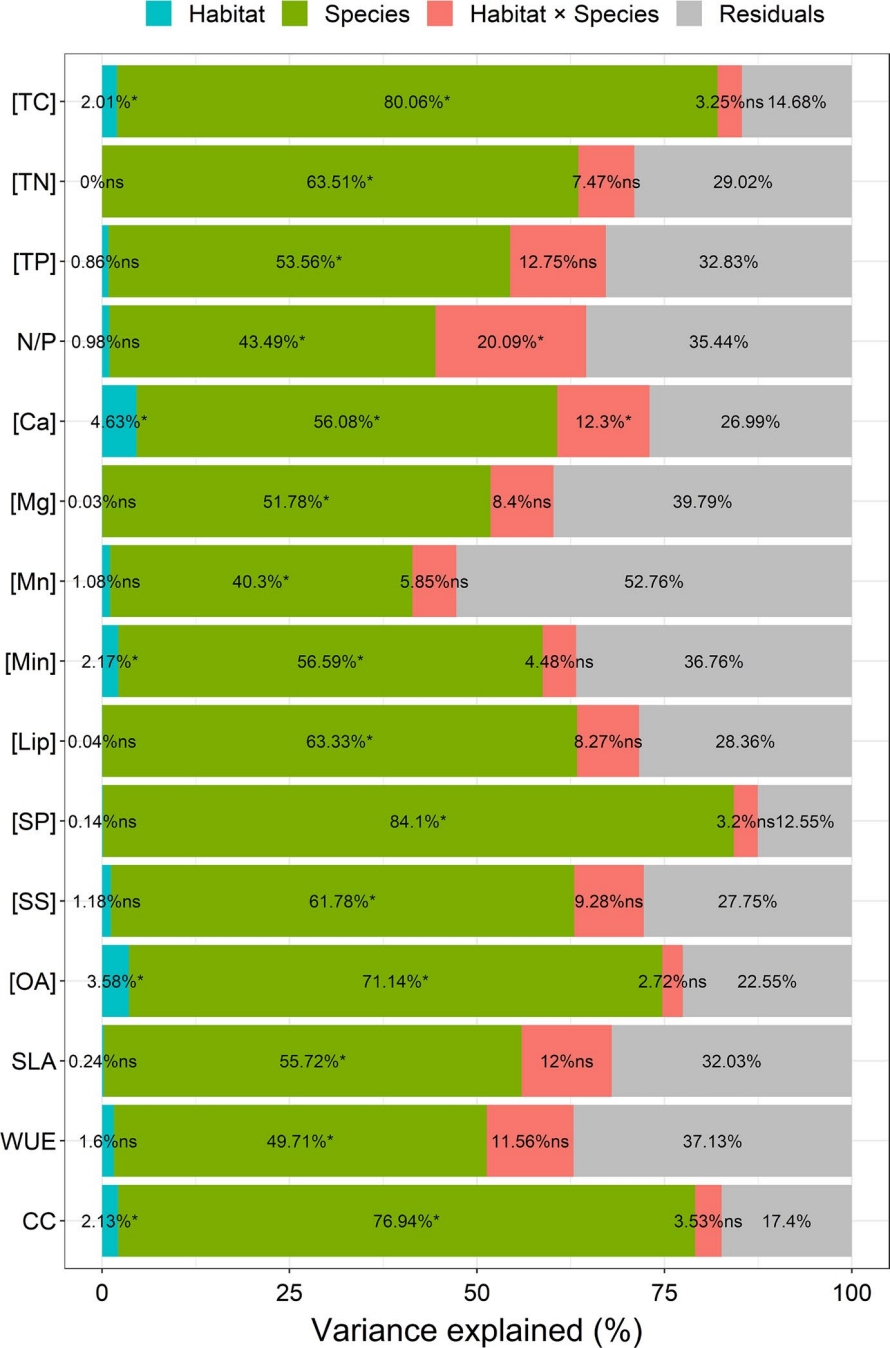
Variance explained for each leaf trait investigated in this study. * and ns indicate significance and nonsignificance, respectively, in two ANOVA analyses at *p* < .05. Abbreviations of all leaf traits are provided in Table 2

**FIGURE 2:**
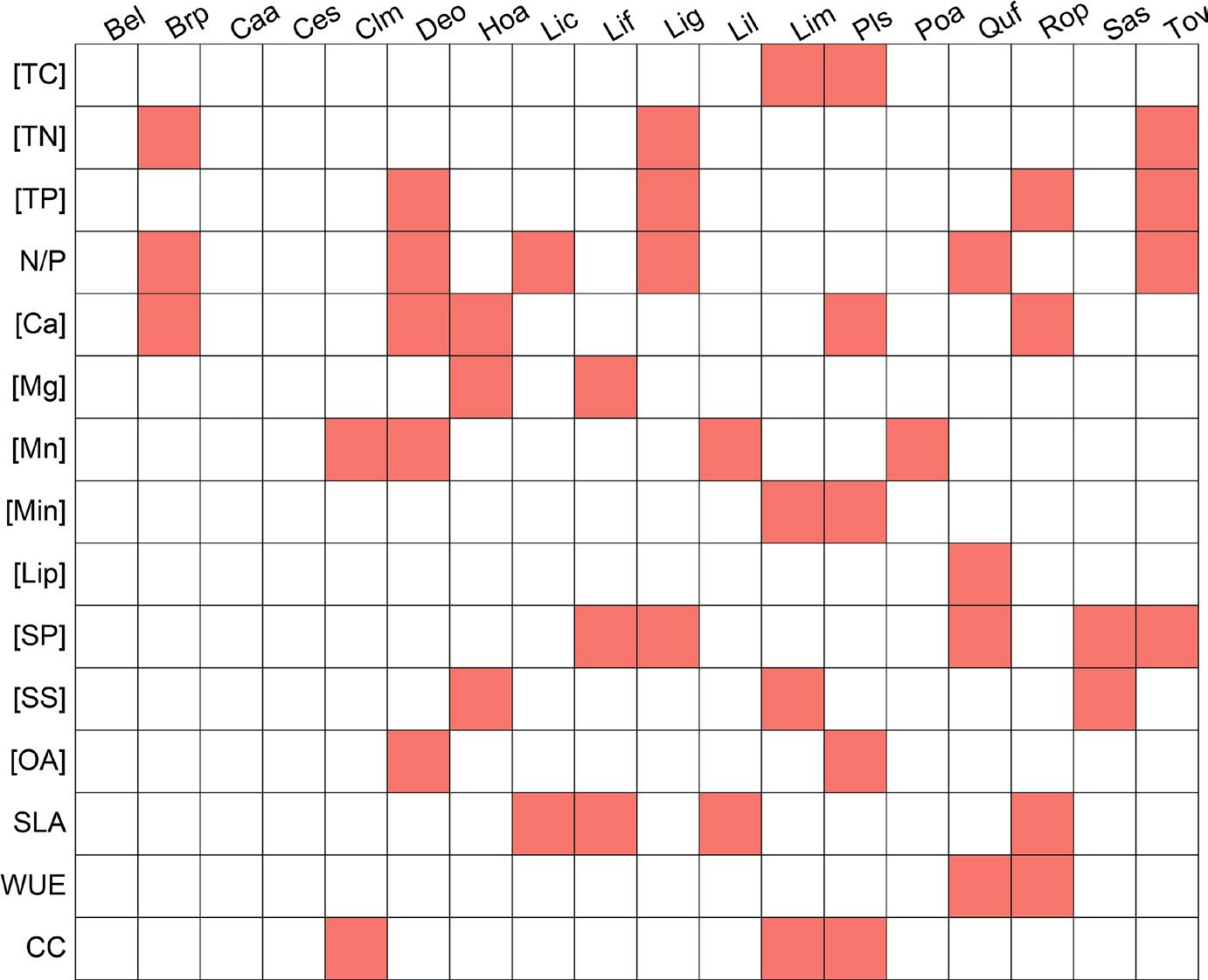
Variations (*chi‐squar*e) of the studied leaf traits within tree species from karst to nonkarst habitats (Kruskal test). Squares with a significant difference (*p* < .05) are filled. Bel, *Betula luminifera*; Brp, *Broussonetia papyrifera*; Caa, *Camptotheca acuminata*; Ces, *Celtis sinensis*; Clm, *Clerodendrum mandarinorum*; Deo, *Debregeasia orientalis*; Hoa, *Hovenia acerba*; Lil, *Ligustrum lucidum*; Lig, *Lindera glauca*; Lif, *Liquidambar formosana*; Lic, *Litsea cubeba*; Lim, *Litsea mollis*; Pls, *Platycarya strobilacea*; Poa, *Populus adenopoda*; Quf, *Quercus fabri*; Rop, *Robinia pseudoacacia*; Sas, *Sapium sebiferum*; Tov, *Toxicodendron vernicifluum*. Abbreviations of all leaf traits are provided in Table 2

**FIGURE 3:**
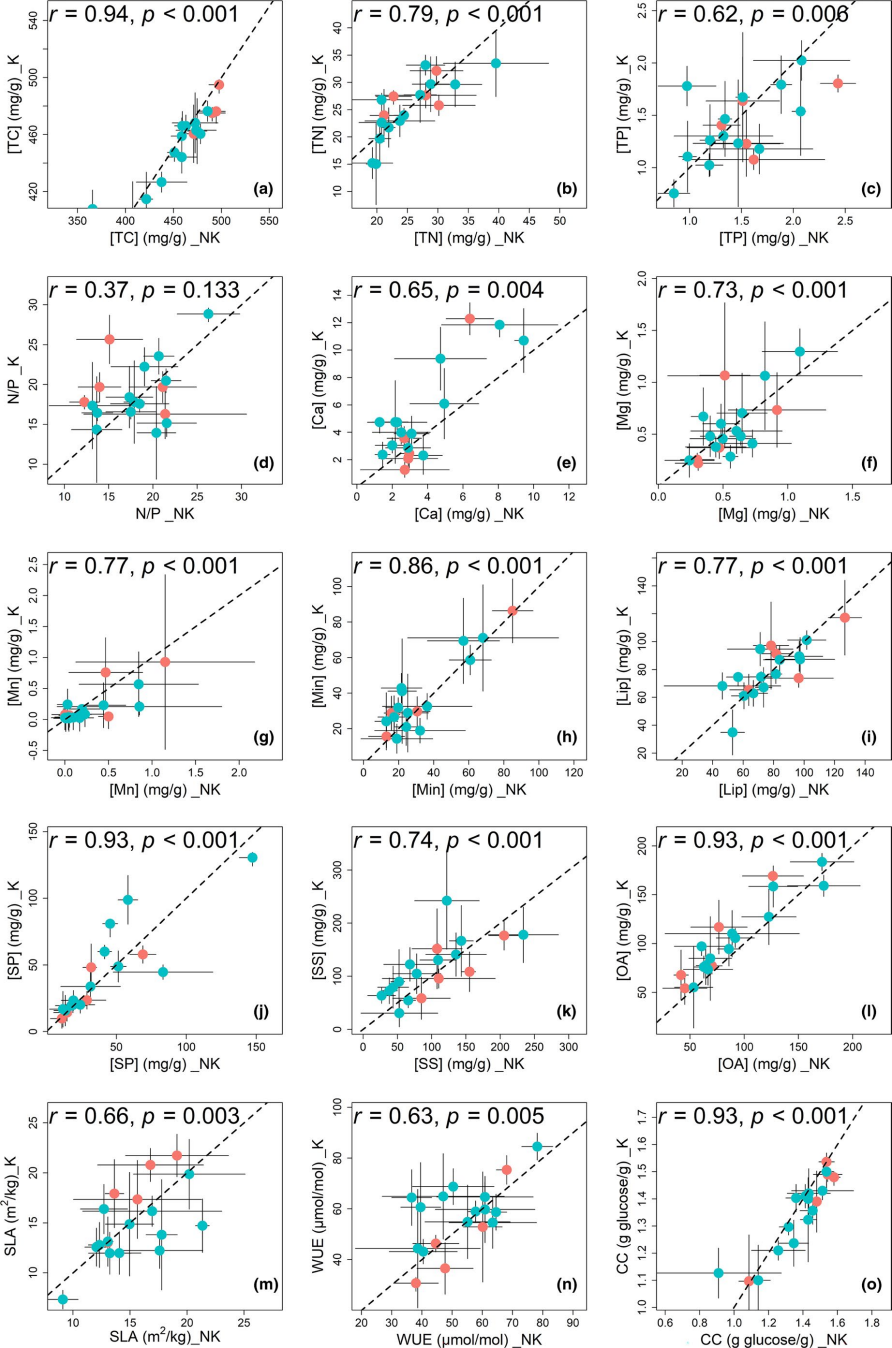
Correlations (Pearson's correlation coefficients, *r*) between the traits of 18 common tree species from karst (K) and nonkarst (NK) habitats. The abbreviations of the tree species are indicated in Table 1. Abbreviation of all leaf traits is provided in Table 2

**FIGURE 4:**
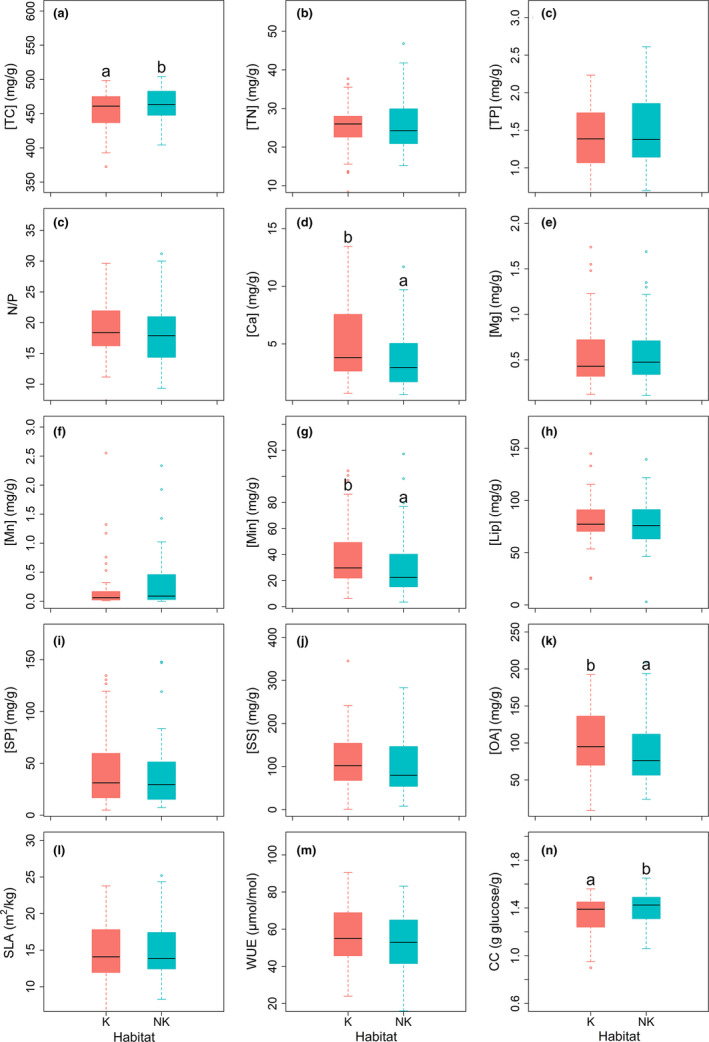
Differences in leaf traits of the dominant plant species between karst and nonkarst habitats. Different lowercase letters indicate significant differences between habitats based on linear mixed effect models (post hoc Tukey test, *p* < .05). The absence of lowercase letters indicates that the effect of habitat was not significant. Boxes in each boxplot show the first and third quartiles and the median; the upper and lower whiskers indicate the largest and smallest values away from 1.5*IQR (interquartile range) of the third quartiles and first quartiles, respectively; black points in each figure are values that fell outside the whiskers. Abbreviation of all leaf traits is provided in Table 2

All the studied leaf traits were affected by species (Figure 1). The variations in the leaf traits were inconsistent among species (Figure 2). Furthermore, five leaf traits ([TC], [Ca], [Min], [OA], and CC) were affected by habitat, and two leaf traits ([Ca] and N/P ratios) were affected by the interaction of habitat and species (Figure 1). We quantitatively assessed the degree of departure from the *y* = *x* line with values in karst habitats plotted against those in nonkarst habitats and found these five leaf traits affected by habitat substantially departed from this line across plant species (Figure 3a, e, h, l, o). In addition, leaf CC and [TC] were significantly lower in karst habitats than in nonkarst habitats, while leaf [Ca], [Min], and [OA] were significantly higher in the former than in the latter (Figure 4a, e, h, l, o).

### 3.2 Effects of habitat on the correlations among leaf traits

**FIGURE 5:**
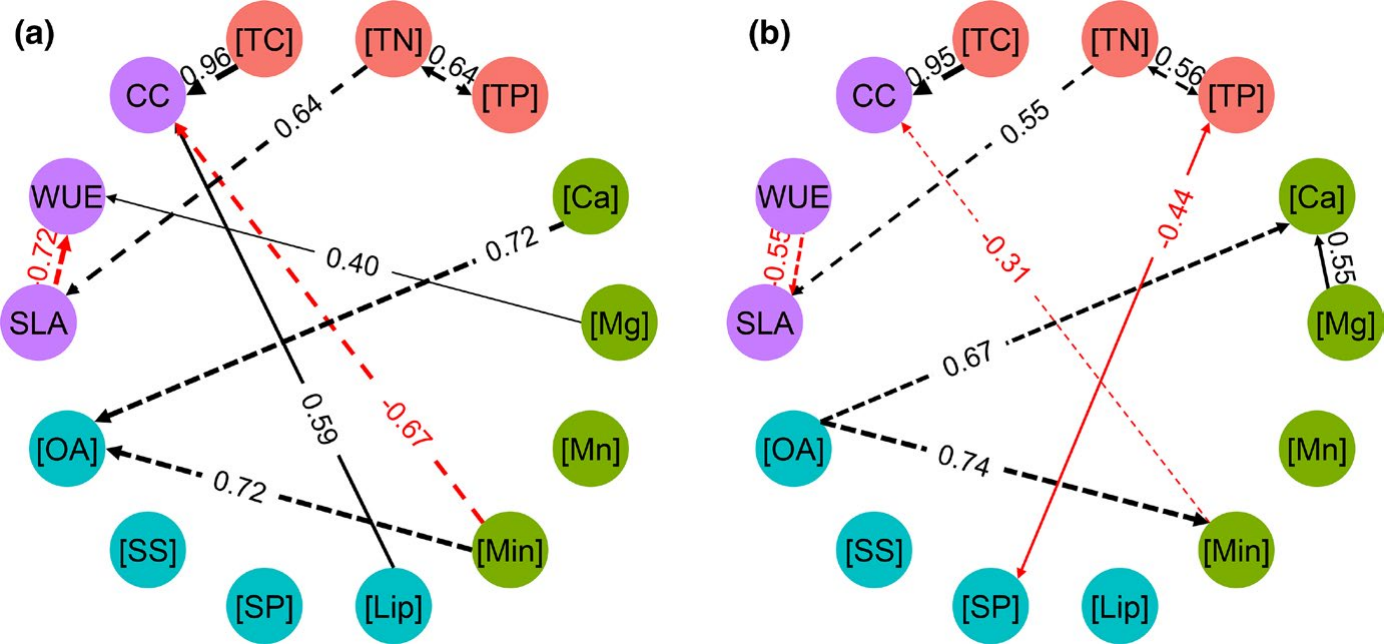
Correlations of the studied leaf traits derived from the idaFast function in karst (a) and nonkarst (b) habitats. The lines (both dashed and solid ones) linking two traits denote significant correlations (*p* < .05, black for positive and red for negative), and the effect size is shown by number close to the line. The dashed and solid lines indicate that correlations are uniform and different, respectively, between both habitats. A missing edge between two traits indicates no causal effects. Abbreviation of all leaf traits is provided in Table 2

The correlations among leaf traits were either habitat‐independent or habitat‐dependent for the common trees (Figure 5a, b). In both habitats, the correlations between [TC] and CC (positive), [TN] and SLA (positive), [OA] and [Min] (positive), [OA] and [Ca] (positive), [Min] and [CC] (negative), [TN] and [TP] (positive), and SLA and WUE (negative) were significant (Figure 5a, b). However, in karst habitats, leaf CC was additionally correlated with leaf [Lip] (positively), and WUE was additionally correlated with leaf [Mg] (positively), which was not found in nonkarst habitats. In nonkarst habitats, leaf [Ca] was positively correlated with leaf [Mg] (Figure 5b).

### 3.3 Pathways via which habitat affected leaf trait variations

**FIGURE 6:**
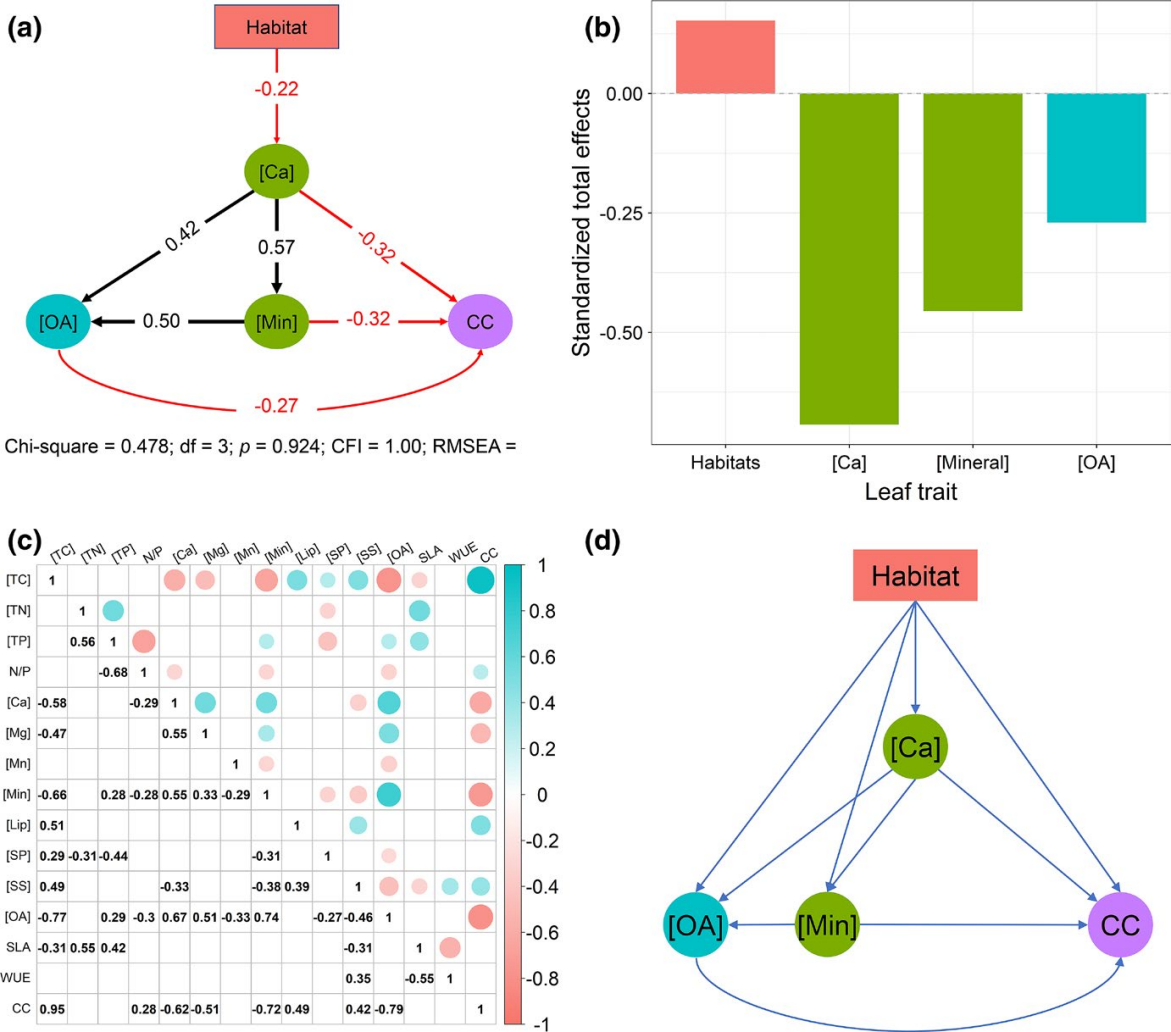
(a) structural equation model (*SEM*) paths of the effects of habitat on leaf traits; (b) the standardized total effects of habitat, leaf [Ca], [Min], and [OA] on leaf construction costs (CC); (c) correlations between leaf traits of 18 dominant tree species. These blank squares indicate that the correlations between leaf traits are nonsignificant (*p* > .05); and (d) the a priori modeling of *SEM*. We removed leaf carbon concentrations when performing *SEM*, because of the high correlation between leaf [TC] and CC (*r* = 0.96, *p* < .001, c). Abbreviation of all leaf traits are provided in Table 2

The effects of habitat on leaf [OA], [Min], and CC were indirect, via affecting leaf [Ca] (Figure 6a). In addition, leaf CC was decreased by both leaf [Ca], [Min], and [OA], but lower for species from karst than those from nonkarst habitats (Figure 6a, b). Furthermore, leaf [Ca] presented a stronger impact on leaf CC than leaf [OA] and [Min] (Figure 6a).

## 4 DISCUSSION

### 4.1 Intraspecific and interspecific variations in leaf traits

A variety of leaf traits reflects adaptation of plants to a specific environment (Hazen et al., 2018), but some exhibit substantial phenotypic plasticity in many plants (Bjorkman et al., 2018; Russo & Kitajima, 2016). In the present study, however, we only observed that habitat had significant effects on plant leaf [TC], [Min], [OA], [Ca], and leaf CC (Figures 1 and 4), but species had significant effects on all leaf traits studied here (Figure 1). The inconsistency between habitats and across the common plants in this study (Figures 1, 2, and 4) segregates the importance of intraspecific (Kraft et al., 2014) and interspecific variations (Albert et al., 2011). Although the dissimilarity of leaf traits decreases competition among coexisting plant species (Kraft et al., 2015), the fine‐scale diversity of hydrogeology, topography, and associated water availability influenced by a monsoon climate (Guo et al., 2017) meets the demand of the dominant plant species for resource acquisition, both in karst and nonkarst habitats.

The differences in soil conditions, especially soil [Ca] and soil water availability, between karst and nonkarst forests (Hao et al., 2015; Wei et al., 2018), may have contributed to the divergence in leaf [Ca], [Min], [OA], [TC], and CC (Figure 4). Our results imply that the different soil properties between karst and nonkarst habitats may have limited impact on most leaf traits in our study, which may be due to similar environmental conditions (Tian et al., 2017), such as adequate soil [TN] and [TP] for plant growth in both habitats in southwestern China (assessed by leaf N/P ratios, karst: 19 ± 4.7; nonkarst: 18 ± 4.8, Geekiyanage et al., 2019), and moderate nutrient retrieval by plant growth in karst habitats (Liu et al., 2015). Part of our first aim of this study is to assess whether leaf traits are affected by habitat and species. We found that five leaf traits were significantly affected by habitat, while all leaf traits were affected by species.

### 4.2 The importance of specific correlations between leaf traits in plant adaptation

Either independent or dependent effects of habitat on the correlations between leaf traits (Figure 5a, b) suggest that combinations of leaf traits, both convergence and divergence, are important for plant adaptation. The leaf traits with habitat‐independent relationships were [TC], [TN], [TP], SLA, CC, [OA], [Ca], [Min], and WUE, implying their importance for resource acquisition and resistance to environmental stress in both karst and nonkarst habitats (Figure 5). Leaf [TN], [TP], and SLA are important traits reflecting plant growth, and CC is associated with plant's carbon budget (Lambers & Poorter, 1992; Lambers et al., 1998; Liu et al., 2016). The positive bidirectional influence between leaf [TN] and [TP] and the positive effect of [TN]/[TP] on plant SLA support the contention that high leaf [TN] and [TP] enhance plant growth (Lambers & Poorter, 1992).

Physiologically, a high WUE tends to be associated with a low SLA (Wellstein et al., 2017), leaf [TN], and [TP] (Prieto et al., 2018; Wright et al., 2005), thus maintaining C, N, and P acquisition and utilization. There are three mechanisms that may explain the negative correlations between SLA and WUE: (a) CO_2_ supply at sites of carboxylation may be decreased due to a longer internal CO_2_ diffusion pathway in thicker leaves (Hultine & Marshall, 2000; Prieto et al., 2018); (b) densely packed mesophyll may reduce the conductance of mesophyll to CO_2_ in thicker leaves (Prieto et al., 2018; Tomás et al., 2013); and (c) more enzymes related to photosynthesis in thicker leaves may increase the demand for CO_2_ (Hultine & Marshall, 2000; Prieto et al., 2018). The negative correlations between leaf nutrients (N and P) and WUE may result from plants enhancing mass flow of nutrients by increasing transpiration and enhancing uptake of mobile nutrients, and plants with high leaf nutrient concentrations increasing stomatal conductance and photosynthetic activity (Field et al., 1983; Prieto et al., 2018). Although there were some habitat‐dependent correlations between leaf traits, for example, positive correlation between leaf [Lip] and CC and between leaf [Mg] and WUE in karst habitats and negative correlations between [TP] and [SP] in nonkarst habitats, we assume that the habitat‐independent correlations of leaf traits are evolutionary outcomes of natural selection, since successful trait combinations are appropriate for plant growth in specific habitat (Ahrens et al., 2020; Firn et al., 2019; Moreira & Pearse, 2017). These habitat‐independent correlations may explain why dominant species grow well in both karst and nonkarst habitats.

**FIGURE 7:**
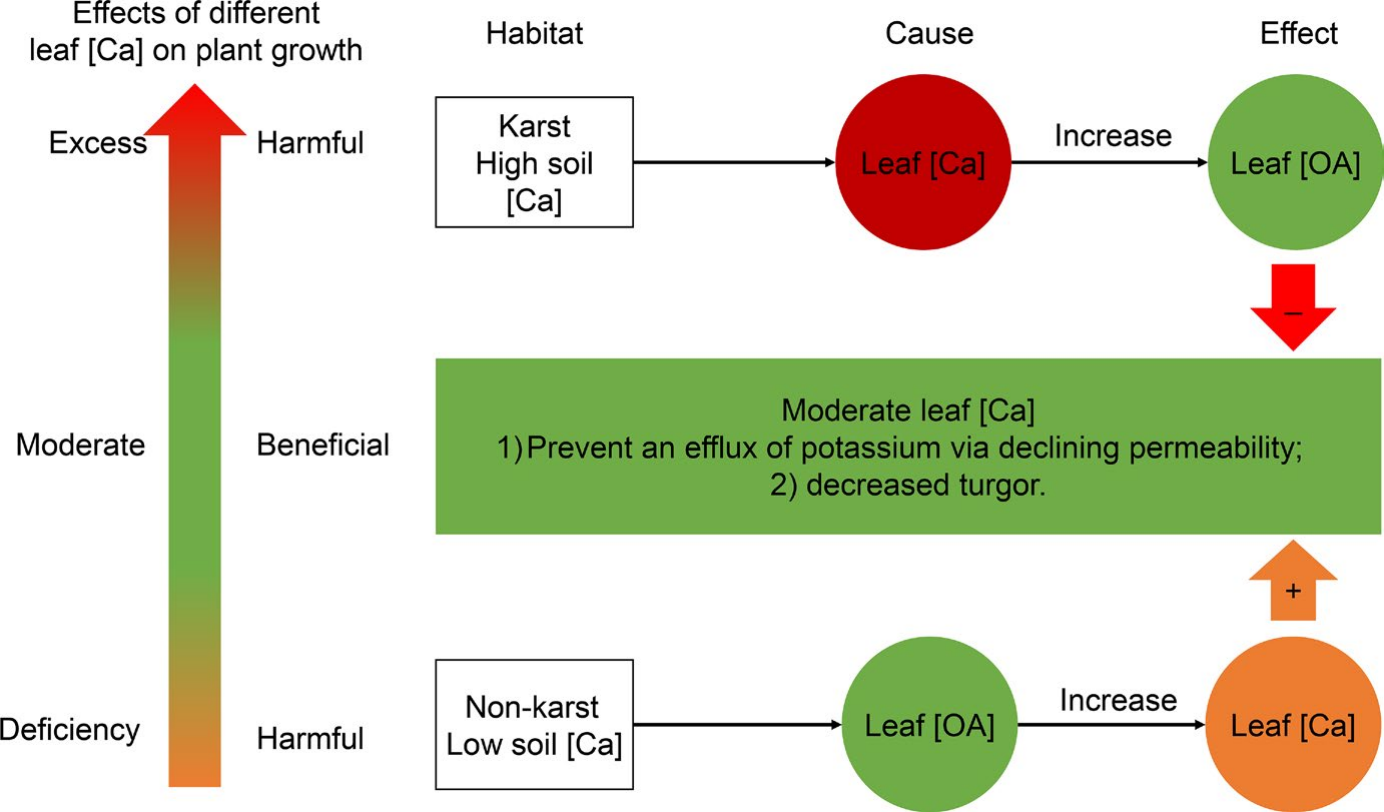
A conceptual model explaining causal effects between the concentrations of leaf calcium ([Ca]) and organic acids ([OA])

The similar effect sizes but opposite directions, between leaf [OA] and leaf [Ca] and [Min] (0.72 vs. 0.67, 0.72 vs. 0.74) in karst vs. nonkarst habitats, suggest the importance of leaf [OA] for plant adaptation through adjusting leaf [Ca] (Triplett et al., 1980). We speculate that both the effects of [Ca] on [OA] in karst habitats and of [OA] on [Ca] in nonkarst habitats are to maintain leaf [Ca] at moderate levels, which can benefit plant growth (Figure 7). Generally, plants growing in karst habitats accumulate OA in leaves to maintain ion balance and to decline the restriction of excess [Ca] on plant growth (Figure 7, White & Broadley, 2003), while those growing in nonkarst habitats need an amount of Ca to maintain normal physiological functions, for example, preventing an efflux of potassium and decreasing turgor (Bressan et al., 1998; Burstrom, 1968). Therefore, plants in nonkarst habitats might enhance Ca via increasing OA in leaves (Figure 7). The role of leaf OA regulating the level of Ca may allow species to dominate in both karst and nonkarst habitats.

### 4.3 High leaf [OA] is a consequence of high leaf [Ca] in karst

Calcium is a plant macronutrient, while abundant Ca has adverse effects, for example, affecting ion uptake by roots (Kinzel, 1983). We found that the effect of habitat on leaf traits was associated with the differences in leaf [Ca] between the habitats (Figure 6), indicating plants are substantially affected by high soil [Ca]. Therefore, the significantly higher leaf [Ca], [Min], and [OA] in karst than in nonkarst habitats (Figure 4) partly reflects the effects of habitat properties, especially high soil [Ca] (Hao et al., 2015; Wei et al., 2018). The high leaf [OA] was likely a consequence of high leaf [Ca] in karst habitats (Triplett et al., 1980). The significantly lower leaf CC ([TC]) in karst habitats than in nonkarst habitats (Figure 4) likely reflects the accumulation of cheap compound (e.g., OA) and minerals (Figure 6a, b; Lambers & Poorter, 1992; Poorter & Bergkotte, 1992). The results suggest that leaf traits of dominant species in karst habitats are mainly affected by leaf [Ca], which may affect plant's adaptability to karst habitats. Considering plant species in this study were sampled from relatively fertile locations in both karst and nonkarst habitats, more studies of plant species only dominating in karst habitats should be conducted to adequately understand how plants adapt to karst habitats.

## 5 CONCLUSIONS

We quantitatively assessed the variations and causal effects of leaf traits of plant species common in both karst and nonkarst habitats. We showed that the variations in leaf traits within and across the common plant species were both divergent and convergent between the habitats, and the correlations between leaf traits were either dependent or independent of habitat. Leaf [OA] was affected by leaf [Ca] and [Min] in karst habitats, while leaf [Ca] and [Min] were affected by leaf [OA] in nonkarst habitats. The high leaf [OA] of dominant species may be associated with decreasing adverse effects of high [Ca] in karst habitats. Our results provide insights into the functioning of plant species common both in karst and nonkarst forests. Further studies are warranted to evaluate the physiological effects of leaf [OA] and [Ca] on plant adaptability in karst habitats.

## CONFLICT OF INTEREST

The authors declare no conflict of interest.

## AUTHOR CONTRIBUTION

**Songbo Tang:** Visualization (equal); Writing‐review & editing (equal). **Jianfeng Liu:** Visualization (equal); Writing‐review & editing (equal). **Hans Lambers:** Writing‐review & editing (equal). **Lingling Zhang:** Methodology (equal); Writing‐review & editing (equal). **Zhanfeng Liu:** Writing‐review & editing (equal). **Yutong Lin:** Investigation (equal); Writing‐review & editing (equal). **Yuanwen Kuang:** Conceptualization (lead); Funding acquisition (lead); Writing‐original draft (equal); Writing‐review & editing (equal).

## Data Availability

Data are available from the Dryad Digital Repository https://doi.org/10.5061/dryad.x95x69pjc (Tang et al., 2021).
